# Optimization of an RNA-Seq Differential Gene Expression Analysis Depending on Biological Replicate Number and Library Size

**DOI:** 10.3389/fpls.2018.00108

**Published:** 2018-02-14

**Authors:** Sophie Lamarre, Pierre Frasse, Mohamed Zouine, Delphine Labourdette, Elise Sainderichin, Guojian Hu, Véronique Le Berre-Anton, Mondher Bouzayen, Elie Maza

**Affiliations:** ^1^LISBP, Centre National de la Recherche Scientifique, INRA, INSA, Université de Toulouse, Toulouse, France; ^2^GBF, Université de Toulouse, INRA, Castanet-Tolosan, France

**Keywords:** transcriptomics, RNA-Seq, biological replicates, library size, differential gene expression analysis, power, false discovery rate, gene ontology enrichment analysis

## Abstract

RNA-Seq is a widely used technology that allows an efficient genome-wide quantification of gene expressions for, for example, differential expression (DE) analysis. After a brief review of the main issues, methods and tools related to the DE analysis of RNA-Seq data, this article focuses on the impact of both the replicate number and library size in such analyses. While the main drawback of previous relevant studies is the lack of generality, we conducted both an analysis of a two-condition experiment (with eight biological replicates per condition) to compare the results with previous benchmark studies, and a meta-analysis of 17 experiments with up to 18 biological conditions, eight biological replicates and 100 million (M) reads per sample. As a global trend, we concluded that the replicate number has a larger impact than the library size on the power of the DE analysis, except for low-expressed genes, for which both parameters seem to have the same impact. Our study also provides new insights for practitioners aiming to enhance their experimental designs. For instance, by analyzing both the sensitivity and specificity of the DE analysis, we showed that the optimal threshold to control the false discovery rate (FDR) is approximately 2^−r^, where r is the replicate number. Furthermore, we showed that the false positive rate (FPR) is rather well controlled by all three studied R packages: *DESeq, DESeq2*, and *edgeR*. We also analyzed the impact of both the replicate number and library size on gene ontology (GO) enrichment analysis. Interestingly, we concluded that increases in the replicate number and library size tend to enhance the sensitivity and specificity, respectively, of the GO analysis. Finally, we recommend to RNA-Seq practitioners the production of a pilot data set to strictly analyze the power of their experimental design, or the use of a public data set, which should be similar to the data set they will obtain. For individuals working on tomato research, on the basis of the meta-analysis, we recommend at least four biological replicates per condition and 20 M reads per sample to be almost sure of obtaining about 1000 DE genes if they exist.

## Introduction

Since its first results were published, RNA-Seq technology has been widely perceived as a revolutionary tool for transcriptomics (Wang Z. et al., 2009). It has become a prevalent technology, allowing an efficient genome-wide relative quantification of gene expression and, in particular, it is the method of choice to find differentially expressed genes between two or more biological conditions of interest. From the beginning, the main issues related to such DE analysis have been pointed out, and many methods and tools have been proposed in the relevant literature. As for any other statistical analysis, one main issue has been finding the probabilistic model that best fits the data, as well as the optimal parameter estimates of this model. Another important issue was the need for normalization of the data to correctly compare two different biological conditions by assessing and erasing all eventual technical and/or biological biases. Last but not least, the practical need to find the optimal number of biological replicates per condition and the optimal library size have also been highlighted in many studies. Here, we introduce these issues and review some widely used methods and tools for DE analysis. This review will help us to choose the most relevant methods and tools to perform DE analyses in the present work.

### The probabilistic model

The problem of finding the best model to fit RNA-Seq data has been tackled recently by Gierlinski et al. (2015). The authors recommend the use of tools based on the negative binomial distribution. These tools include *edgeR, DESeq, DESeq2, Cuffdiff*, *Cuffdiff 2*, and *baySeq* (Anders and Huber, 2010; Hardcastle and Kelly, 2010; Robinson et al., 2010; Trapnell et al., 2012, 2013; Love et al., 2014). There are also some non-parametric methods that can be used as alternatives when the data do not seem to fit the negative binomial law, but these methods are less often used and usually require a higher replicate number to perform equally well (Spies and Ciaudo, 2015).

### The normalization method

When the RNA-Seq technology was first introduced, Wang H. et al. (2009) and other pioneers thought that it could be used without sophisticated normalization methods. On the contrary, Bullard et al. (2010) have demonstrated the high impact of the normalization procedure on the DE analysis. Many of the normalization methods proposed in the literature are based on the correction of biases or artifacts directly related to the RNA-Seq technology, such as transcript lengths and sequencing depths, non-uniformity of read distributions along transcripts and strong sample-specific GC-content effect (Mortazavi et al., 2008; Oshlack and Wakefield, 2009; Zenoni et al., 2010; Risso et al., 2011; Roberts et al., 2011; Tarazona et al., 2011; Hansen et al., 2012). The relative size of transcriptomes in the studied biological conditions is another crucial, not technical, bias affecting DE analysis. Such a bias has been addressed by Robinson and Oshlack (2010) and Anders and Huber (2010), who proposed, respectively, the trimmed mean of *M*-values (TMM) and the relative log expression (RLE) normalization methods (Anders et al., 2013). Moreover, it has been shown that both the TMM and RLE methods give similar results and outperform other existing normalization methods in DE analysis (Dillies et al., 2013; Maza et al., 2013). Nevertheless, Chen et al. (2016) have shown that spike-in controls are compulsory for the normalization of some particular RNA-Seq experiments, but these situations are not predominant in practice, and fall outside the scope of our article.

### Benchmark articles on replicates and depth

To our knowledge, only a few recent articles have aimed to exclusively and deeply analyze the impact of the replicate number and library size (or depth) on a DE analysis. Three studies conclude that increasing the number of biological replicates is globally a more efficient strategy than increasing the library sizes, in order to enhance the power and the false discovery rate (FDR) of a DE analysis (Ching et al., 2014; Liu et al., 2014; Schurch et al., 2016). Nevertheless, these three studies also give specific results concerning their analyzed data sets. Liu et al. (2014) and Ching et al. (2014) conclude that, with their analyzed data sets, a library size of respectively 10 and 20 M reads per sample is the minimum threshold for an effective DE analysis. Schurch et al. (2016) give more general recommendations based on their single data set study; they recommend at least six biological replicates per condition in general, and at least 12 replicates to identify the majority of DE genes. In addition, some authors provide tools to estimate an optimal number of biological replicates per condition based on a pilot data set of the given experimental design or on the specification of desired coefficients of variation (CV) or dispersions of the future results (Busby et al., 2013; Hart et al., 2013; Li et al., 2013; Ching et al., 2014; Wu et al., 2015).

### Some methods and tools performing DE analysis

With the rise of the RNA-Seq technology, many methods and tools have appeared for DE analysis (Table 1 gives an almost comprehensive list of 29 R packages or tools dedicated to DE analysis, and summarizes information above concerning the used probabilistic model and normalization method). Consequently, many comparison studies have been carried out, but there is not yet a gold standard method. Moreover, many comparison studies highlight that no single method outperforms others in all circumstances (Rapaport et al., 2013; Soneson and Delorenzi, 2013; Zhang et al., 2014; Seyednasrollah et al., 2015). Nevertheless, it seems that some tools are particularly appropriate. Soneson and Delorenzi (2013) concluded that, for large sample sizes, the *limma* methods perform well, as does the non-parametric *SAMseq* tool. Seyednasrollah et al. (2015) concluded that *limma* and *DESeq* methods are the safest choices with a small number of replicates, that *edgeR* gives very variable results, and that *SAMseq* suffers from a lack of power. Also, with many replicates, the choice of the method and/or tool is less critical (unless for *NOISeq* and *Cuffdiff 2*). Rapaport et al. (2013) concluded that *DESeq, edgeR*, and *baySeq* have superior specificity and sensitivity, and seem to outperform the *limma* and *PoissonSeq* methods. The worst method seems to be *Cuffdiff*. Burden et al. (2014) concluded that the *QuasiSeq* tool achieves a low FDR providing the number of replicates in each condition is at least 4. The next best performing packages are *edgeR* and *DESeq2*. In other studies, both *edgeR* and *DESeq* seem to give similar and correct or better results (Kvam et al., 2012; Robles et al., 2012; Zhang et al., 2014; Conesa et al., 2016; Lin et al., 2016).

**Table 1:** Information on 29 R packages, methods, or pipelines, for DE analysis of RNA-Seq data: the number of citations of the article introducing the method (until October 2017, extracted from All Databases of Web of Science^a^), the used probabilistic model, the default normalization method, and whether or not a Bayesian approach is considered.

**R-package or method**	**References**	**December, 2013**	**January, 2015**	**January, 2016**	**January, 2017**	**October, 2017**	**October, 2017 (%)**	**Distribution**	**Normalization**	**Bayesian**
*edgeR*	Robinson et al., 2010	430	982	1,854	3,040	4,450	22.00	Negative binomial	TMM	No
*Cufflinks* (*Cuffdiff*)	Trapnell et al., 2010	861	1,648	2,446	3,300	4,283	21.17	Poisson	FPKM (geometric)	Yes
*DESeq*	Anders and Huber, 2010	607	1,395	2,299	3,167	4,157	20.55	Negative binomial	RLE	No
*DESeq2*	Love et al., 2014			83	282	1,899	9.39	Negative binomial	RLE	Yes
*vst* or *QN* + *limma*	Ritchie et al., 2015					1,276	6.31	Gaussian	vst or QN	Yes
*Cuffdiff 2*	Trapnell et al., 2013			421	699	950	4.70	Beta negative binomial	Geometric	No
*DEGSeq*	Wang et al., 2010	178	297	458	636	850	4.20	Poisson	Total count	No
*voom* + *limma*	Law et al., 2014					493	2.44	Gaussian	log-CPM	Yes
*NOISeq*	Tarazona et al., 2011	65	164	263	377	473	2.34	Non-parametric	CPM	No
*baySeq*	Hardcastle and Kelly, 2010	72	109	178	232	302	1.49	Negative binomial	Total count	Yes
*EBSeq*	Leng et al., 2013	5	31	93	170	270	1.33	Negative binomial	RLE	Yes
*Myrna*	Langmead et al., 2010	57	88	112	117	149	0.74	Poisson or Gaussian	3rd quartile	No
*SAMseq*	Li and Tibshirani, 2013	0	22	52	91	129	0.64	Non-parametric	Trimmed total count	No
*GFOLD*	Feng et al., 2012			41	73	93	0.46	Hierarchical Poisson	RLE	Yes
*PoissonSeq*	Li et al., 2012	4	19	43	63	88	0.44	Poisson	Trimmed total count	No
*DSS*	Wu et al., 2013			31	44	61	0.30	Gamma-Poisson	3rd quartile	Yes
*BBSeq*	Zhou et al., 2011	15	21	30	40	50	0.25	Beta-binomial	Total count	No
*QuasiSeq*	Lund et al., 2012					42	0.21	Negative binomial	3rd quartile	No
*TSPM*	Auer and Doerge, 2011	8	12	16	26	39	0.19	Two-stage Poisson	Total count	No
*ShrinkSeq*	Wiel et al., 2013	5	14	18	28	33	0.16	Zero-inflated negative binomial	None	Yes
*GENE-counter*	Cumbie et al., 2011	9	14	20	26	30	0.15	Negative binomial	Total count	No
*NBPSeq*	Di et al., 2011	11	14	21	23	28	0.14	Negative binomial	Total count	No
*sSeq*	Yu et al., 2013					27	0.13	Negative binomial	RLE	No
*Polyfit*	Burden et al., 2014					15	0.07	Negative binomial	RLE	No
*NPEBseq*	Bi and Davuluri, 2013	0	4	11	12	14	0.07	Gamma-Poisson	TMM	Yes
*BMDE*	Lee et al., 2011	4	5	8	8	10	0.05	Binomial (position-level)	Total count	Yes
*LFCseq*	Lin et al., 2014					6	0.03	Non-parametric	Trimmed total count	No
*CEDER*	Wan and Sun, 2012	0	1	2	4	5	0.02	Negative binomial	RLE	No
*ShrinkBayes*	van de Wiel et al., 2014			1	3	5	0.02	Zero-inflated negative binomial	None	Yes

a http://apps.webofknowledge.com

Table 1 also provides the number of citations of articles introducing above cited tools. We notice that *edgeR* appears first with 22% of citations, followed by *Cufflinks* (21%, but we do not know the number of citations that are exclusively due to *Cuffdiff*), *DESeq* (20%), *DESeq2* (9%), and then, all other tools below 6%.

Finally, the choice of the methods we used in this article for DE analyses was done by looking at considerations above and comparison studies, but also considering that our *in silico* approaches were extremely time consuming and that no comprehensive study was able. We then decided to compare the following four widely used methods: *DESeq, DESeq2, edgeR* with the *exact test* and *edgeR* with the *GLM*. Moreover, considering again comparison studies above, these four methods seem to give similar results, and we then arbitrarily chose only one for the most time consuming analyses.

In the present article, we aim to study the impact of the replicate number and library size on the DE analysis of an RNA-Seq experiment involving the tomato fruit model (*Solanum lycopersicum*). Our study rely on two data sources. On the one hand, we analyzed a two-condition data set with eight biological replications per condition and 20 M reads per sample from the Tomato Ovary Gene Expression (TOGE) project. On the other hand, in order to give more general recommendations, we performed a meta-analysis with all the RNA-Seq experiments available on the *TomExpress* platform, i.e., 16 projects, 124 biological conditions, and 348 biological samples (Zouine et al., 2017).

## Materials and methods

### Plant materials and experimental design of the TOGE project

Tomato plants (*Solanum lycopersicum L. cv. Micro-Tom*) were grown in a culture chamber set as follows: a 14 h/10 h day/night cycle, a 25°C/20°C day/night temperature dynamic, 80% relative humidity, and 250 μmol·m^−2^·s^−1^ light intensity.

The ovaries (including style and stigma) and the developing young fruits were collected as samples. Ovaries were picked on the first day of flower opening (anthesis stage) and set as 0 days post-anthesis (DPA). Developing young fruits were picked 4 days after this natural pollination stage and set as 4 DPA. Sampling procedures were mainly as described in Wang H. et al. (2009). Eight biological replicates were performed for each studied condition (0 DPA and 4 DPA). For each biological replicate, more than 50 ovaries were pooled from 25 plants.

Total RNA was isolated from 200 and 500 mg, respectively, of ovary and young fruit powders (*TRIzol Reagent, Life Technologies*). After DNase treatment (*DNA-free Kit, Life Technologies*), the total RNA quantity and quality were assayed using an *Agilent 2100 Bioanalyzer* (*Agilent Technologies*). Only RNA with an RNA integrity number (RIN) above 8.0 was used for sequencing. The RNA libraries were constructed as described in the *Illumina TruSeq Stranded mRNA Guide*. mRNA was sequenced in a *HiSeq 2500 sequencing system* with 2 × 125 bp paired-end sequences (*Illumina HiSeq SBS Kit v4*).

### RNA-Seq data mapping and quantification of the TOGE data

A quality check of the raw sequences was made with *FastQC*^1^. Trimming was performed with PRINSEQ (version 0.20.3) with the option –*trim right*. Reads were aligned with a spliced alignment tool to the genome of *Solanum lycopersicum* (SL2.40.22 and ITAG.2.3 GFF3 annotation file) with *TopHat 2* (version 2.0.14) (Kim et al., 2013). On average, between 80 and 90% of the reads were aligned to the reference genome.

We randomly down-sampled the reads to generate data sets of 2.5, 5, 7.5, 10, 15, and 20 M reads using the python script *get_subset.py* before alignment on the reference genome^2^. We used *SAMtools* view option –*s* for down-sampling the reads after the alignment on the reference genome (Li et al., 2009).

Raw counts were generated on each gene by using *HTSeq-count* (version 0.6.1p1) with the option –*stranded* = *reverse* (Anders et al., 2015). Moreover, since reads can overlap one or more features, we used the mode intersection-non-empty, which guarantees the highest number of assignments.

### DE analysis of the TOGE data

All DE analyses of the Number of DE genes (section Number of DE Genes of the TOGE Data) and Power (section Power Analysis of the TOGE Data) were performed with R software (version 3.2.0) and the dedicated *edgeR* package (version 3.6.8) (Robinson et al., 2010; R Core Team, 2015). No filtering was applied. The TMM normalization method was performed to normalize the counts among the different samples (Robinson and Oshlack, 2010; Maza et al., 2013; Maza, 2016). The dispersion parameter was estimated in two different ways, depending on the number of replicates, to enable a more robust estimation: if the number of replicates was less than or equal to 4, we estimated the dispersion by the *CommonDisp* function; otherwise, the dispersion was estimated using the *TagwiseDisp* function (Robinson et al., 2010). In order to detect significantly DE genes, we used the *exactTest* function. A gene was declared as significantly DE if its adjusted *p*-value (controlling the FDR) was less than 0.05 (Benjamini and Hochberg, 1995).

To analyze the impact of the number of replicates and the library size on the DE analysis, we built 45 data sets for each number of replicates among two, three, four, five, six, and seven replicates, and each library size among 2.5, 5, 7.5, 10, 15, and 20 M reads. Each replicate was randomly chosen without replacement among the eight samples for each condition. We then analyzed 36 combinations of replicate number and library size, from the smallest with two replicates and 2.5 M reads to the largest with seven replicates and 20 M reads. Then, for each combination, we had 45 DE gene lists, and we computed the median of the two studied indicators: the number of DE genes and the estimated power. Obviously, for eight replicates and each library size, we only had one data set and then one indicator. For the calculation of the power, we needed a reference list of DE genes. For this purpose, we chose the DE genes that were found with all available information (i.e., with eight replicates and 20 M reads) and with a very stringent adjusted *p*-value = 0.0001. Then, for a given DE gene list, the power was calculated by the ratio of the number of true DE genes (i.e., genes that are considered to be DE and that belong to the reference list above) to the total number of genes in the previous reference list (see also the section “Sensitivity and Specificity” below).

Moreover, to calculate the stability of each indicator, we retained, for each combination of replicate number and library size, the DE genes that were common to all 45 data sets. We then calculated both indicators for this new list of DE genes.

Finally, to analyze the impact of the gene expression level on the studied indicators, the gene set was divided into three parts: genes with low counts, genes with medium counts and genes with high counts, i.e., those with a logCPM (counts per million reads) less than the first quartile, between the first and the third quartile, and higher than the third quartile, respectively. Both indicators were then calculated and presented for both low and high expression levels.

### Gene ontology (GO) analysis of the TOGE data

We performed an enrichment analysis with the *goseq* R package (version 1.20.0) (Young et al., 2010). As tomato is not referenced in *goseq*, we manually built the list of GO biological process (BP) identifiers and lengths of genes. The tomato GO terms were downloaded from the *UniProtKB* database^3^. The *goseq* tool is suitable for RNA-Seq enrichment analysis, since it allows an adjustment for gene selection thanks to differences in gene lengths, which are known to affect the variance of gene expression estimates. BP categories with *p*-values less than 0.05 were considered to be significantly enriched. For each combination of depth and replicate number, lists of the common BP categories obtained for the 45 essays were extracted and analyzed (in the same way as described above for the stability of the number of DE genes and the power).

### Sensitivity and specificity of the TOGE data

For a given DE analysis method, the sensitivity (or true positive rate, TPR) and the specificity (or true negative rate, TNR) are defined as follows. The TPR is the number of significantly DE genes that are true DE genes, divided by the total number of true DE genes. The TNR is the number of non-significant DE genes that are true non-DE genes, divided by the total number of true non-DE genes. Moreover, we have that specificity = TNR = 1 – FPR (false positive rate). The FPR is then equal to the number of significantly DE genes that are true non-DE genes, divided by the total number of true non-DE genes.

The four DE analysis methods studied here were carried out using the R software environment (version 3.1.3) (R Core Team, 2015) and the corresponding packages *DESeq* (version 1.18.0), *DESeq2* (version 1.6.3), and *edgeR* (version 3.8.6) with both *GLM* method and the *exact test* method. All four methods have been applied with the corresponding default normalization methods and parameterizations. All these packages can be uploaded from the Bioconductor website (Gentleman et al., 2004).

As described above, the calculation of TPR, TNR, and FPR values requires the knowledge of the list of all true DE genes between our two biological conditions, which is obviously not the case in practice. In order to estimate these true DE genes, we performed a prior DE analysis for each method with the whole data set, i.e., eight replicates per condition and all available reads. Moreover, for this prior analysis, we chose a stringent threshold equal to 0.001 to control the FDR (Benjamini and Hochberg, 1995). We then obtained four lists of genes that estimated the unknown list of truly DE genes for the four DE analysis methods. Using a specific estimated list of truly DE genes for each method enables a relatively objective measure of the performance of each method (Schurch et al., 2016).

### DE meta-analysis of *TomExpress* and TOGE data

A DE meta-analysis was performed for all the biological conditions of the *TomExpress* and *TOGE* data sets. For each pair of biological conditions, a DE analysis was done with the *DESeq2* R package with default settings and a threshold of 0.05 to control the FDR.

For a given condition, simulated replicates were carried out by a convex linear combination of existing replicates with uniform random coefficients. For this purpose, we used conditions that had two or more replicates. Then, for each simulated replicate, raw counts were randomly carried out with a multinomial distribution with probabilities given by the true observed probabilities of genes, and with library sizes of 5, 10, 15, 20, and 25 M reads. These calculations aim at simulating pseudo-replicates that have almost the same characteristics (means and variances) as the true ones.

## Results

### Number of DE genes of the TOGE data

**Figure 1:**
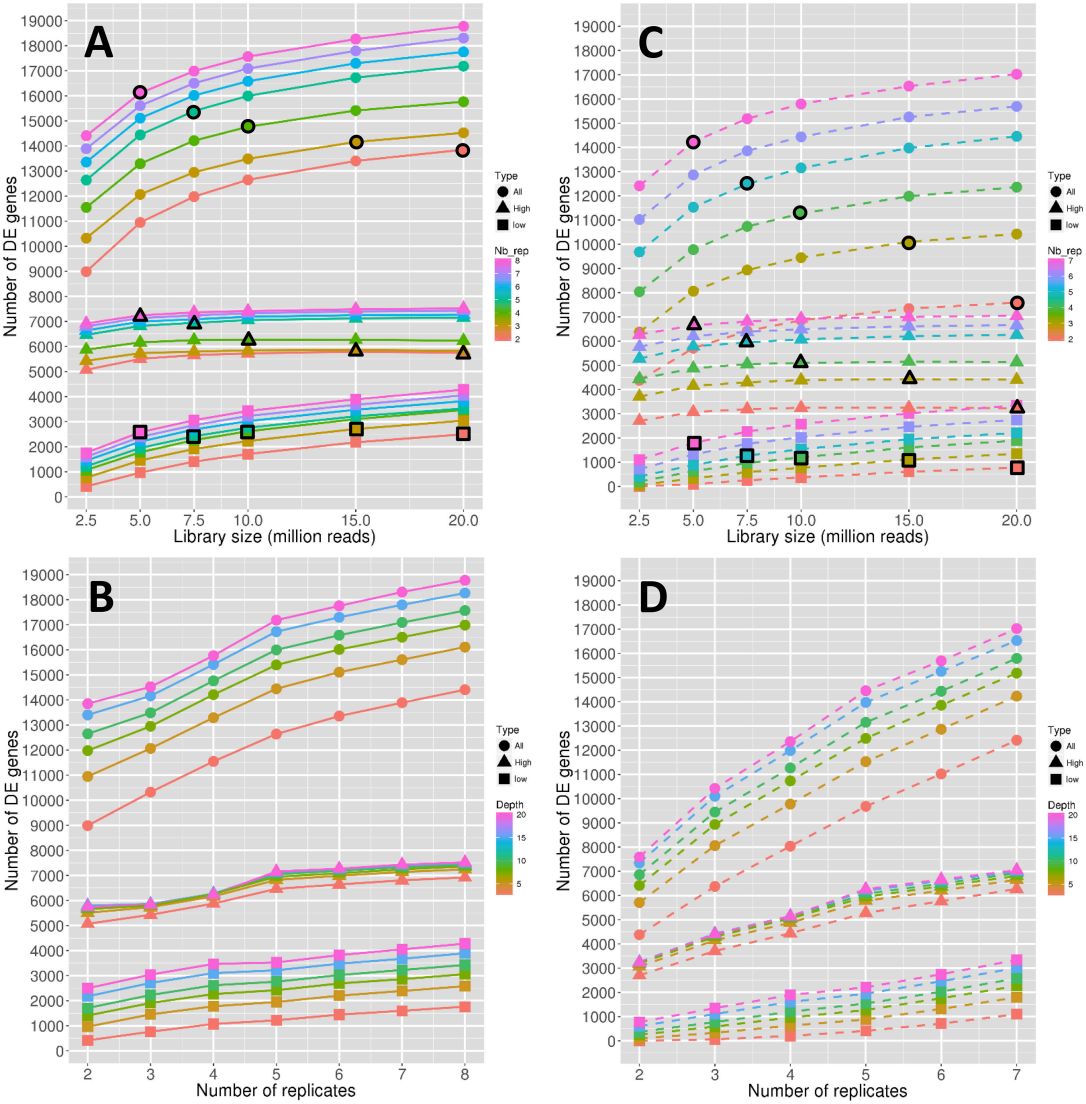
Number of DE genes depending on the depth **(A)** and on the replicate number **(B)**. Stability of the number of DE genes depending on the depth **(C)** and on the replicate number **(D)**. Symbol colors correspond to the replicate numbers for **(A,C)** and to the library sizes for **(B,D)**. Colored circles, triangles, and squares represent, respectively the values obtained with all genes, high expressed genes, and low expressed genes. Colored circles, triangles, and squares that are surrounded with a black line correspond to combinations of library sizes and replicate numbers with a total amount of 40 M reads approximately.

The number of significantly DE genes obtained between conditions 0 DPA (flower before pollination) and 4 DPA (flower after pollination) is shown in Figure 1. More precisely, Figures 1A,B show the evolution of the number of DE genes depending on the library size and the replicate number, respectively. In the same way, Figures 1C,D focus on the stability of the number of DE genes, depending also on the library size and the replicate number. Note that the *number* of DE genes is hereafter defined as the median number of DE genes obtained for 45 DE analyses, and, in the same way, the *stability* of the number of DE genes is defined as the number of common DE genes obtained for the 45 DE analyses (see Materials and Methods).

All the observed curves in Figures 1A,B show a more or less increasing dynamic, which clearly reflects that both the depth and the replicate number are important in the detection of DE genes. Nevertheless, by comparing dynamics of genes with low and high expressions, it seems that the former are more impacted than the latter by the increase in depth, as curves representing low-expressed genes increase faster than curves representing high-expressed genes (Figure 1A). The increase of the replicate number seems to have the same impact on both expression levels (Figure 1B). Moreover, for all genes, the rate of increase seems to diminish after 10 M reads (Figure 1A) or five replicates (Figure 1B). Nevertheless, this phenomenon seems to be less intense for low-expressed genes (Figure 1B).

To determine whether the library size or the replicate number has a relatively higher impact on the number of DE genes, we needed to compare combinations of these two parameters that shared the same total amount of reads. This comparison is shown in Figure 1A, where symbols with a black border represent a combination with a total amount of about 40 M reads. Moreover, the three curves in Figure 1A, depicted by black border circles, triangles and squares, can be interpreted as follows: a constant curve implies an equal effect of the depth and replicate number parameters, a decreasing curve implies a higher impact of the replicate number, and an increasing curve implies a higher impact of the library size. We can then clearly see in Figure 1A that, for all genes, the number of replicates has a higher impact on the number of DE genes than the library size. By looking solely at high-expressed genes, we can see that the replicate number is again more important than the library size. On the contrary, low-expressed genes seem to be equally impacted by the library size and the replicate number.

The stability of the number of DE genes represented in Figures 1C,D is an indicator quantifying the dispersion of the number of DE genes: a higher stability reflects a lower variability of the declared DE genes (see Materials and Methods). Biologically speaking, the stability is perhaps a more important indicator than the number of DE genes, in that it reflects the variability of the DE analysis method. Globally, we can see from Figures 1C,D that the stability has lower values than the number of DE genes described above. For instance, for three replicates and 15 M reads, we have about 14,000 DE genes and a stability of 10,000 DE genes, which means that about 30% of the declared DE genes are specific to the biological replicates. Moreover, for all genes, it appears that the increase rate of the stability curves depending on the replicate number (Figure 1D) is higher than that of the curves of the number of DE genes (Figure 1B), while it remains almost equal for the curves depending on the library sizes (Figures 1A,C). This indicates that the gain of robust DE genes, i.e., DE genes that do not depend on the biological variability, is higher when adding replicates than when increasing the library size. For high-expressed genes, this dynamic is more intense than for low-expressed genes, which can still gain robustness by increasing the library size.

Comparing the effects of library size and replicate number on stability (by looking as above at symbols with a black border in Figure 1C representing a constant total number of reads), we can see that the effect of the replicate number on stability is greater than that on the number of DE genes (curves decrease faster than in Figure 1A). Moreover, even low-expressed genes seem to have a slightly decreasing curve.

### Power analysis of the TOGE data

**Figure 2:**
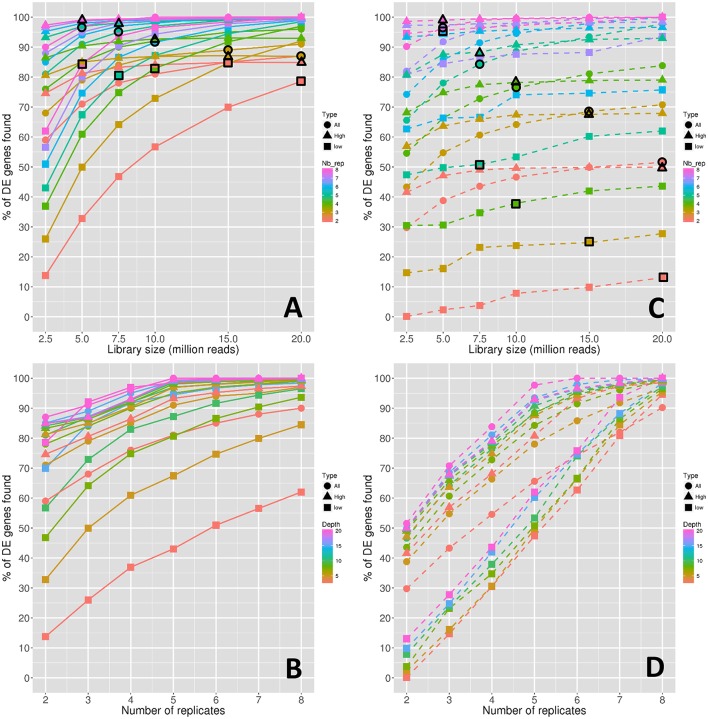
Power of the DE analyses depending on the depth **(A)** and on the replicate number **(B)**. Stability of the power depending on the depth **(C)** and on the replicate number **(D)**. Symbol colors correspond to the replicate numbers for **(A,C)** and to the library sizes for **(B,D)**. Colored circles, triangles, and squares represent respectively the values obtained with all genes, high expressed genes, and low expressed genes. Colored circles, triangles, and squares that are surrounded with a black line correspond to combinations of library sizes and replicate numbers with a total amount of 40 M reads approximately.

Figure 2 shows the power of the DE analyses performed between conditions 0 DPA and 4 DPA. More precisely, in the same way as in Figure 1, Figures 2A,B show the evolution of the power depending on the library size and the replicate number, respectively. Figures 2C,D show the stability of the power depending on the library size and the replicate number, respectively. Note that the *power* is hereafter defined as the median percentage of true DE genes obtained for the 45 DE analyses, and, in the same way, the *stability* of the power is defined as the power corresponding to the common true DE genes obtained for the 45 DE analyses (see Materials and Methods).

Clearly, in the same way as for the number of DE genes discussed in the previous section, both the power and its stability increased with both the library size and the replicate number. Moreover, for all genes, the increase rate diminishes after 10 M reads for all curves of Figure 2A, and after five replicates for all curves of Figure 2B, except for the curve with 5 M reads. For high-expressed genes, the power curves are globally higher than for all genes but have the same trend. On the contrary, for low-expressed genes, the power curves are lower than for all genes, but their rate of increase decreases more slowly.

The large impact of both library size and replicate number on the power for low-expressed genes can be confirmed by looking at black border symbols in Figure 2A, which correspond to a constant number of reads. Indeed, for all genes and for high-expressed genes, the replicate number has a higher impact on power than the library size, whereas low-expressed genes seem to be equally impacted by both parameters.

In Figures 2C,D, it can be clearly seen that the power stability is much lower than the power. For instance, for three replicates and 15 M reads, the power is around 88% (Figure 2A) and the power stability is around 68% (Figure 2C), underlining that approximately 20% of the founded true DE genes depend on the biological variability. This effect is much more intense for low-expressed genes.

Finally, even more than for the number of DE genes discussed above in Figure 1, the impact of the replicate number on the power stability is higher than the impact of the library size. Indeed, by looking at black border symbols in Figure 2C corresponding to a constant number of reads, it is clear that the decrease rates of all gene curves, and of both low- and high-expressed gene curves, are much more intense than those of the corresponding curves in Figure 2A.

### Sensitivity and specificity of the TOGE data

Here, we analyze the sensitivity and the specificity of four classical and widely used DE analysis methods: the first one developed in the *DESeq* R package, the second from the *DESeq2* R package, and two others from the *edgeR* R package, namely the *GLM* and the *exact test* methods (see Materials and Methods).

Calculations of sensitivity (TPR) and 1−specificity (FPR) depend on the knowledge of the true list of DE genes for the biological conditions in question. This list is obviously not known in practice, and we therefore need to estimate it. In a study of the optimal replicate number, this kind of estimation is classically done by considering that true DE genes can be found with all data information, i.e., all replicates, and a very stringent threshold to control the FDR (see Materials and Methods). We then obtain the following estimated numbers of so-called true DE genes: 15110 with *DESeq*, 17010 with *DESeq2*, 17115 with *edgeR GLM*, and 16943 with *edgeR exact test*. The number of commonly declared true DE genes in the four methods is equal to 15046, which corresponds to approximately 86% of genes that have been declared true DE with at least one method. Only the *DESeq* method seems to be more stringent, since the other three methods all declare 94% of these same genes as true DE. The Venn diagram of these results is shown in Figure S1. Globally, the estimated true DE genes are almost the same in all four methods. We used these estimated true DE genes to estimate TPR and FPR values (see Materials and Methods).

**Figure 3:**
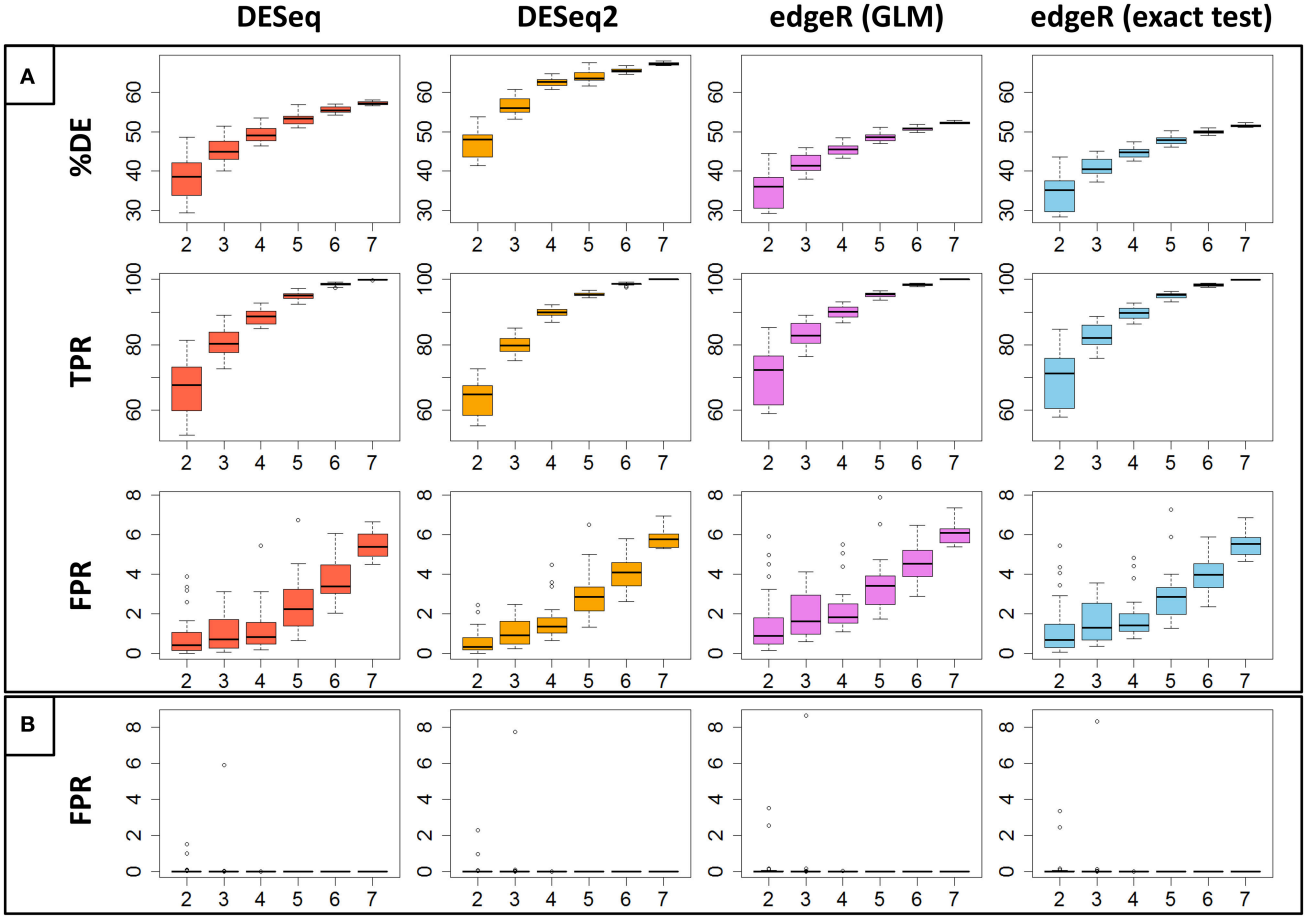
**(A)** Represents the percentage of DE genes (%DE), the estimated True Positive Rate (TPR) and the estimated False Positive Rate (FPR) for each of the four studied methods, depending on the replicate number. Each estimation has been randomly carried out 30 times and the boxplot of these repetitions has been drawn. **(B)** Represents, in the same way, FPR values calculated between replicates of the same biological condition.

Figure 3A represents, for each of the four studied methods, the percentage of significant DE genes (%DE) and the estimated TPR and FPR values depending on the number of replicates (from 2 to 7) with a fixed threshold of 0.01 to control the FDR. Moreover, each value estimation randomly repeated 30 times for each method and each number of replicates, a boxplot of these values is shown in the figure. We can clearly see in this figure that the %DE globally increases for all methods. *DESeq2* seems to catch more DE genes for any number of replicates; *edgeR GLM* and *edgeR exact test* seem to have the same behavior. It is also clear that the TPR increases for all four methods, with decreasing variability: 90% of all DE genes are found with four replicates, increasing to almost 100% with seven replicates, although the gain is minimal with five and more replicates; *edgeR GLM* and *edgeR exact test* have slightly higher TPR values for a reduced replicate number (two or three replicates), but these values are more dispersed. A less obvious result is that FPR values also increase with the number of replicates, from about 1% with two replicates to about 6% with seven replicates. We then have a negative impact of the increasing number of replicates on FPR. This trend was also seen for both low- and high-expressed genes, depending on both replicate number and library size (see Figure S2).

An alternative way to estimate the FPR for a given DE analysis method consists of performing the DE analysis between replicates of the same biological condition (Schurch et al., 2016). Clearly, in that case, all DE genes are false discoveries. Figure 3B represents, for each of the four studied methods, the estimation of the FPR depending on the number of replicates with, as before, a fixed threshold of 0.01 to control the FDR. We again randomly repeated the measure 30 times for each method and each replicate number. We can easily see that, in this case, all methods control the FDR very well. Indeed, all methods have only three values that are higher than 1% (for two and three replicates). Moreover, with five or more replicates, all FPR estimations are equal to 0 (see Figure S3 for a zoomed version of Figure 3B). This result is contradictory with Schurch et al. (2016), for which *DESeq2* gives higher FPR values than *DESeq, edgeR GLM* and *edgeR exact test*.

### Estimation of the optimal threshold controlling the FDR from receiver operating characteristic (ROC) curves depending on replicate number

**Figure 4:**
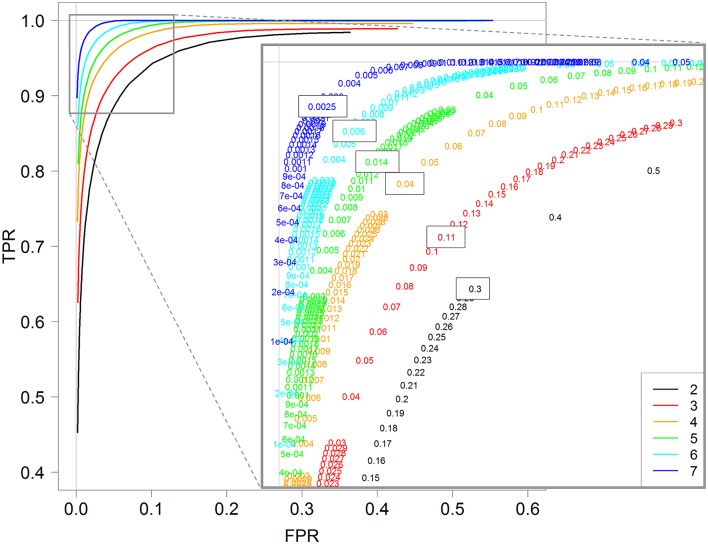
ROC curves for 2, 3, 4, 5, 6, and 7 replicates. Each curve is calculated by varying the *padj* parameter of the *DESeq* function (*DESeq2* package) between 0 and 1. A zoom of the top left corner of the ROC curves is also provided with the detailed *padj* values.

In the above section, TPR and FPR were calculated for a fixed value of the threshold controlling the FDR (0.01). We now investigate the impact of this threshold on both TPR and FPR values by calculating them with different threshold values in the interval [0,1]. Figure 4 shows the ROC curves obtained for each replicate number from 2 to 7 with the *DESeq2* method. Recall here that a ROC curve is preferred to another one when its values are higher; we can then see clearly that increasing the replicate number gives better ROC curves, with an optimal curve corresponding to the curve with seven replicates (blue curve).

More interestingly, we can also see that the optimal threshold values of these ROC curves, i.e., the black-boxed values of the zoomed graph of Figure 4, decrease when the replicate number increases: 0.3 for two replicates, 0.11 for three replicates, 0.04 for four replicates, and so on, and eventually 0.0025 for seven replicates. Hence, for instance, an arbitrary choice of 5% for a DE analysis with three replicates per condition would not be optimal, in the sense that with a threshold of about 10% we would have many more true positive genes and only slightly more false positive ones (see red values on the zoomed graph of Figure 4). We here recall that the multiple testing correction procedure is based, among others, on the number of performed tests, and that our analysis does not modify this approach, but only highlights the relationship between the replicate number and the optimal threshold controlling FDR (which can be chosen by the user).

Furthermore, almost identical results can be obtained for the other three methods: *DESeq, edgeR GLM* and *edgeR exact test* (see Figures S4–S6). Moreover, for all four methods, the optimal value of the threshold to control the FDR is approximately equal to 2^−r^, where r is the number of replicates: 0.25 for two replicates, 0.12 for three replicates, 0.06 for four replicates, and so on, and finally 0.007 for seven replicates (see Figure S7 for the estimation).

**Figure 5:**
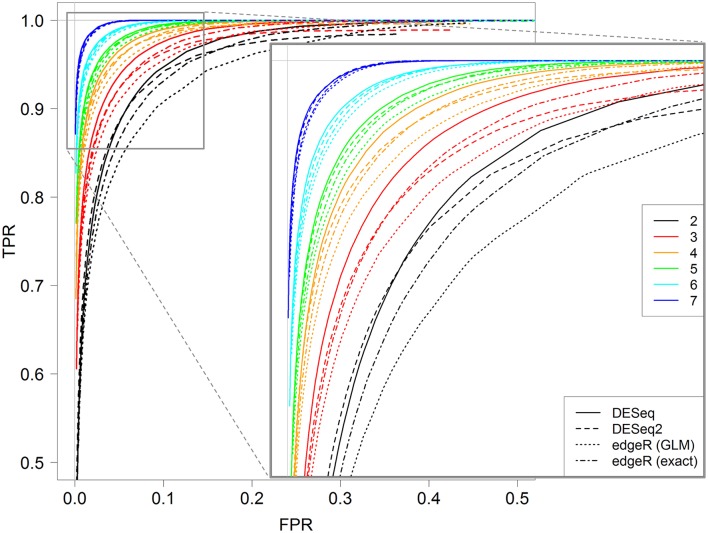
ROC curves for all four studied methods, *DESeq, DESeq2, edgeR* (GLM) and *edgeR* (exact), for 2, 3, 4, 5, 6, and 7 replicates. Each curve is calculated by varying the parameter controlling the FDR with the corresponding method, between 0 and 1. A zoomed graph of the upper left corner of the ROC curves is also shown.

Figure 5 shows ROC curves for all four methods for 2–7 replicates. It can be seen that, for each replicate number, the *DESeq* method seems to give optimal results; indeed, the corresponding continuous black line is almost always above all other lines. Moreover, *DESeq2* and *edgeR exact test* give similar results, and *edgeR GLM* gives the worst ones. Nevertheless, for a higher number of replicates (more than five), these differences tend to be less intense.

### GO enrichment analysis of the TOGE data

**Figure 6:**
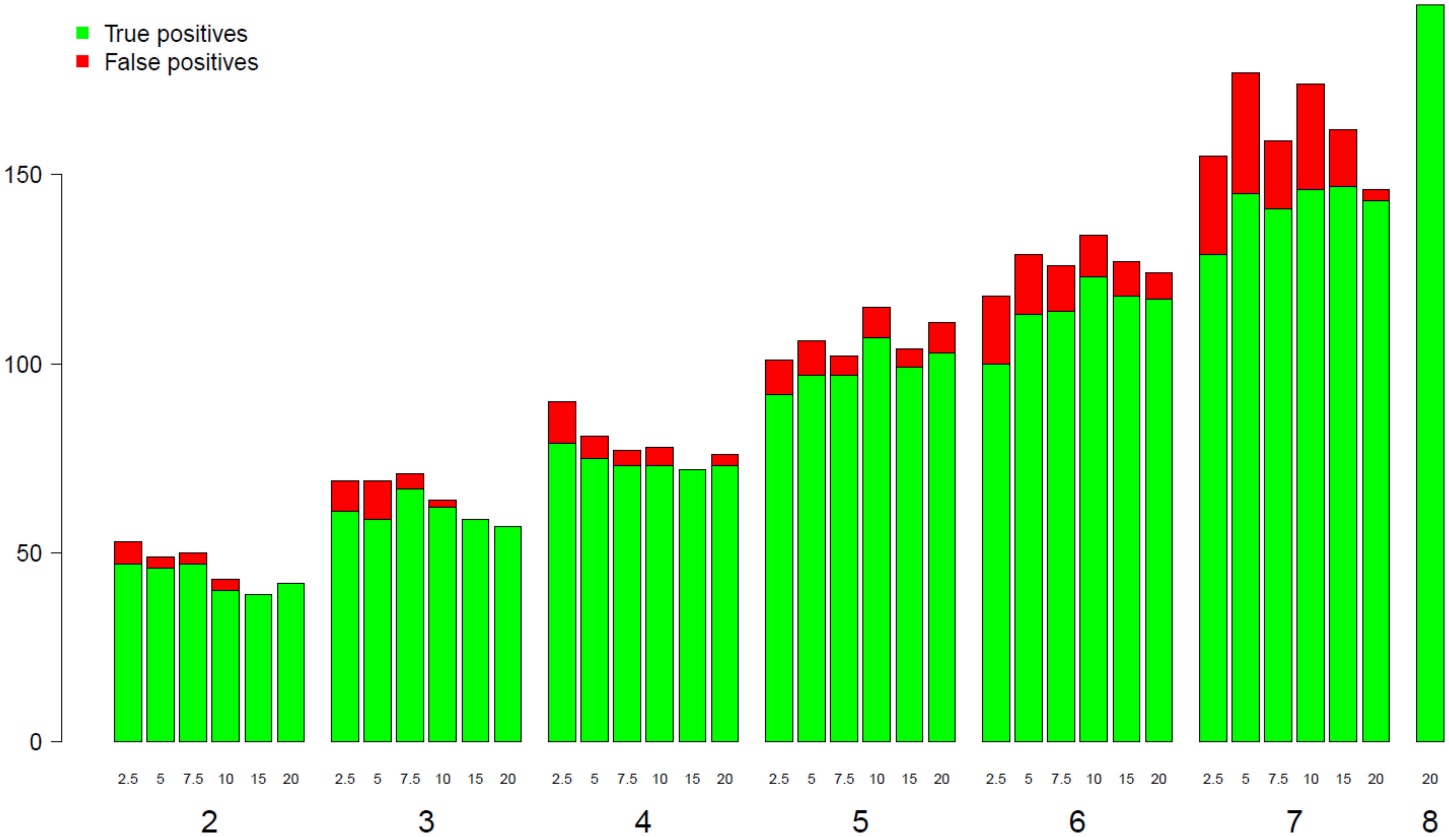
Number of true and false positive BP categories from GO analyses (y-axis) depending on the replicate number (x-axis). The eighth bar corresponding to 8 replicates has been chosen as a reference.

To assess the impact of both the library size and the replicate number on the detection of GO BP categories, we conducted a GO enrichment analysis at each different combination of depth and replicate number using the *goseq* R package. Figure 6 shows the evolution of the number of both true and false positive categories depending on library size and replicate number. The green bar for eight replicates corresponds to the reference gene list obtained with all possible information (i.e., all replicates and all reads). As shown in Figure 6, for a given replicate number, the increase of the library size from 2.5 to 20 M reads does not significantly impact the number of enriched BP categories, but seems to slightly decrease the number of false positive ones. However, when increasing the replicate number from 2 to 7, the number of enriched BP categories was almost tripled. These results suggest that the enrichment stability of the BP categories depends more on the biological replicate number than on the library size.

### DE meta-analysis of *TomExpress* and TOGE data

A DE meta-analysis has been performed with all the *TomExpress*^4^ data plus the TOGE data described above. *TomExpress* is an RNA-Seq platform that was developed to provide the tomato community with a dedicated browser and tools for public RNA-Seq data handling. Our analysis was performed on 17 projects, each containing from two to 18 biological conditions with up to eight biological replicates and 100 M reads. Two kinds of analyses were performed: a description of all DE analyses performed in each project, and a simulation of all possible DE analyses of all pairwise biological conditions of all projects for different replicate numbers and library sizes. The results of these two analyses are described hereafter.

**Figure 7:**
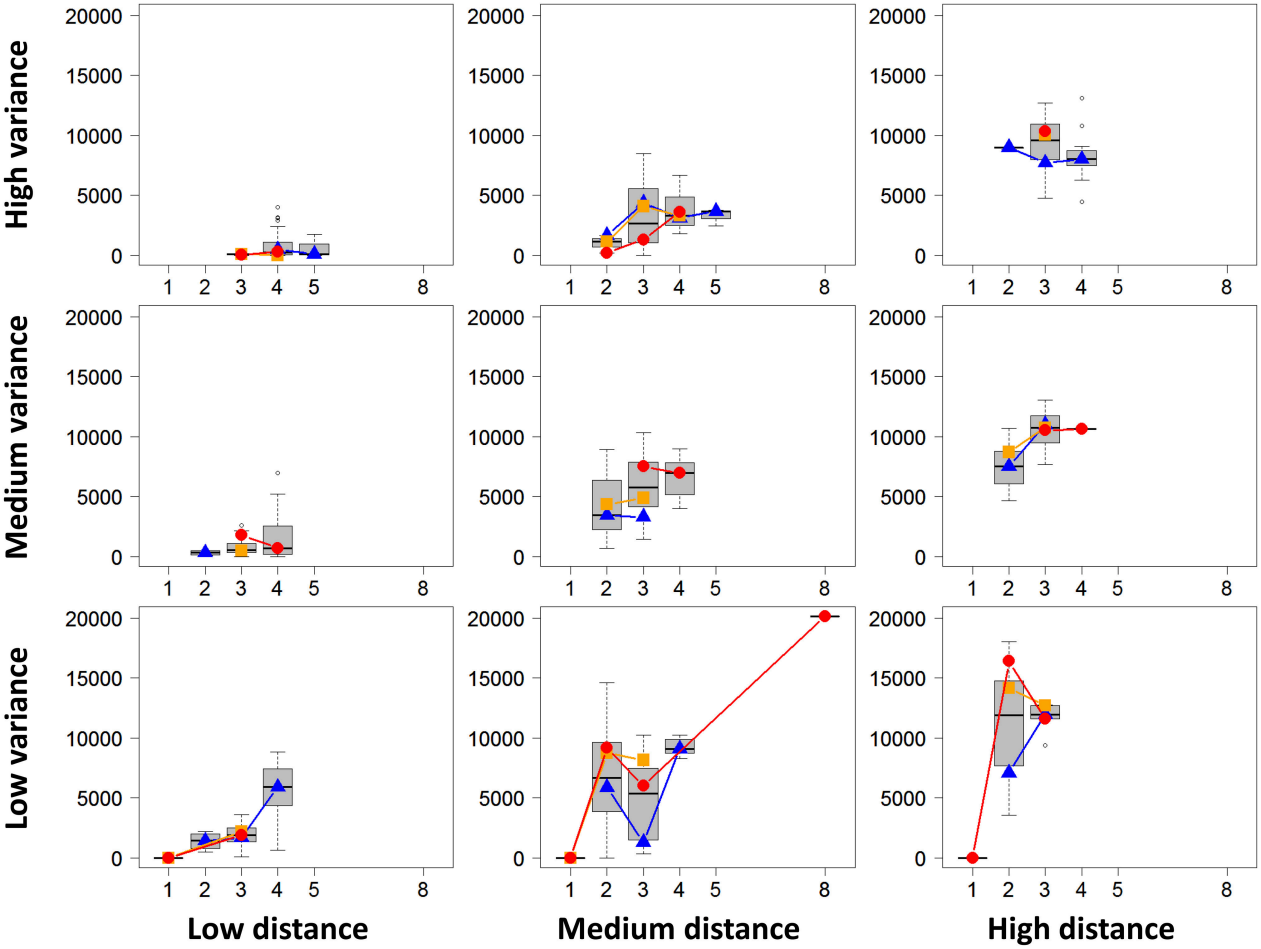
Number of DE genes for each pair of conditions (y-axis) depending on replicate number per condition (x-axis) are represented (by mean of boxplots) for given levels of distances between conditions and of variances of these conditions (low, medium, and high levels). Blue triangles, orange squares, and red circles represent the median numbers of DE genes for respectively low, medium and high library sizes.

For the first descriptive analysis and for each project, we performed all possible DE analyses of all pairwise biological conditions. We then obtained a total of 604 pairwise comparisons. For each DE analysis, we extracted the following characteristics: the number of DE genes, the rounded mean number of biological replicates per biological condition, the mean library size per biological replicate, the mean absolute distance between two biological condition means, and the mean of all gene variances in both biological conditions. Figure 7 summarizes the obtained values for each distance and variance level using boxplots of the number of DE genes depending on the replicate number. Figure 7 also shows, for each distance and variance level, the median number of DE genes for low, medium and high sequencing depth (corresponding, respectively, to the blue, orange, and red dots and lines). We can clearly observe that a higher distance or a lower variance tend to globally increase the number of DE genes. Moreover, as expected, for given distance and variance levels, an increasing number of replicates or increasing sequencing depth also tend to increase the number of DE genes. Nevertheless, the number of DE genes does not only depend on these four parameters, even if it is deeply linked to them. Obviously, the number of DE genes also depends on the biological conditions themselves, which contribute to the huge variability of the number of DE genes in Figure 7.

**Figure 8:**
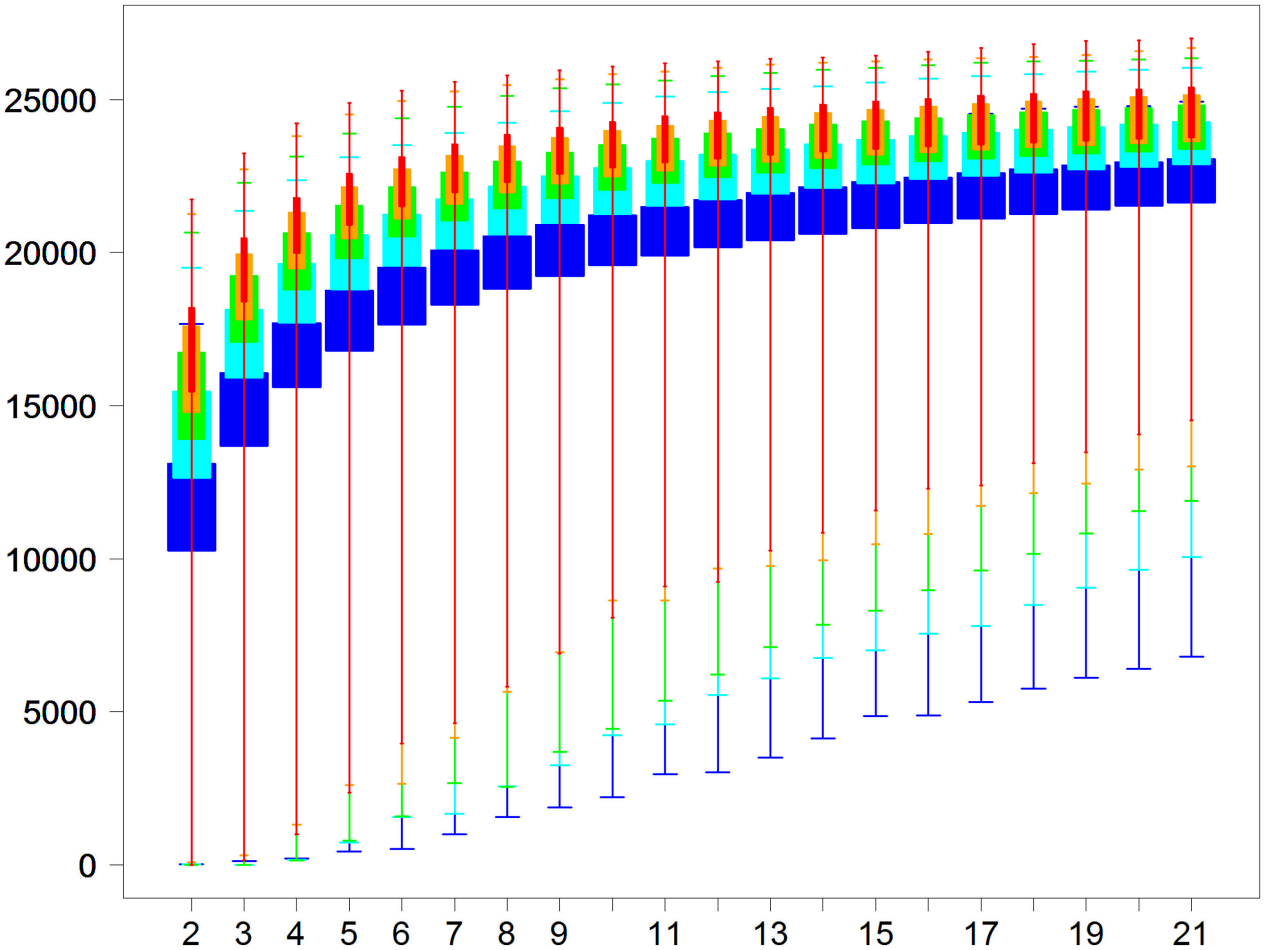
Number of DE genes for each pair of conditions (y-axis) depending on the replicate number per condition (x-axis) for five library sizes (5, 10, 15, 20, and 25 million reads for, respectively, blue, cyan, green, orange, and red boxplots).

In a second analysis, we performed DE analyses of all pairs of biological conditions, no matter which project they came from. Moreover, for each biological condition, we simulated between two and 21 replicates with library sizes of 5, 10, 15, 20, and 25 M reads (we repeated each simulation three times). The DE analysis was then performed to extract the number of DE genes with a threshold of 0.05 to control the FDR (see Materials and Methods). We finally obtained 5565 pairs of biological conditions × 20 different numbers of replicates × 5 different sizes of libraries × 3 repetitions = 1,752,975 pairwise DE analyses. Boxplots of the number of DE genes are shown in Figure 8, depending on the sequencing depth and on the replicate number. By looking at the minimal number of DE genes of each boxplot in Figure 8, it can be seen that we need at least four replicates and 20 M reads to be almost sure of obtaining a significant number of DE genes, i.e., about 1000 DE genes (minimum of the red boxplot). Obviously, these 1000 DE genes roughly correspond to the minimum of what could be found *in silico*, and, moreover, only other experimental approaches (as qPCR analyses) will be able to validate the differential expressed genes. Then, to obtain almost the same number of DE genes, no matter which conditions are studied, we would need about five or six replicates with 10 and 15 M reads, respectively, and about seven replicates with only 5 M reads. We can also see from Figure 8 that, globally, the number of new DE genes tends to be minimal after 10 replicates.

## Discussion

In the present work, we have conducted a thorough analysis of the impact of both replicate number and library size on an RNA-Seq DE analysis. In this discussion, we will compare our results to those obtained by Ching et al. (2014), Liu et al. (2014) and Schurch et al. (2016), who are, to our knowledge, the only authors that exclusively and deeply address these questions. After reviewing these three benchmark articles, we can conclude that their main drawback is the lack of generality. Indeed, their analyses were performed on, respectively, one data set with two conditions, six data sets (from mouse and human tissues) with between six and 129 conditions, and one data set with 48 conditions. Clearly, their conclusions cannot be easily generalized. In regards to our study, on the one hand, we performed a study on a single data set (the TOGE data set), the results of which will be compared with those of the three benchmark articles described above. On the other hand, we performed a meta-analysis on 17 projects, 126 biological conditions, and 364 biological samples of the tomato fruit model, leading then to more general results.

As did the three benchmark articles, our study concludes that an increase in the replicate number or the library size increases the number of significantly DE genes and the power. However, Liu et al. (2014) found that the increase in the replicate number had a higher impact than the increase of the library size on both the number of DE genes and the power for all gene expression levels. On the contrary, and consistent with Ching et al. (2014), we showed that this impact is slightly less important for low-expressed genes; more precisely, these genes are equally impacted by the increase in the replicate number and the increase in the library size in terms of gain of number of DE genes and power.

All three reference studies and ours show that the curves of number of DE genes and power depending on the library size or on the replicate number reach a plateau after a given value. Nevertheless, it appears that this value is different from one study to another, from one data set to another, between 5 and 20 M reads, and between three and 25 replicates. This result shows, as is emphasized by Liu et al. (2014) in their conclusion, the inability of a single study to give generalizable results, and the need for cross-validation analyses comparing the results of several studies. Surprisingly, Schurch et al. (2016) give general recommendations based on their single data set study. For instance, they recommend at least six biological replicates per condition in general, and at least 12 replicates per condition if identifying the majority of all DE genes is important. In our opinion, these recommendations should be nuanced.

As a novelty, we have introduced the notion of stability of the number of DE genes and power. These two indicators are defined, respectively, as the number of DE genes and power calculated with the common list of DE genes obtained with all simulated samples with given parameters. The stability is then a better biological indicator for the number of DE genes or power. From our results, we can observe very little stability of the power for low-expressed genes, which shows that the list of DE genes is highly related to the used samples. For example, with three replicates and 15 M reads, we have a power of about 85% and a stability of the power of about 25%. For stability indicators of both the number of DE genes and the power, we showed that the increase of the replicate number has a higher impact than the increase of the library size for all gene expression levels. This impact is much higher than for the number of DE genes and the power.

We also estimated the FPR, i.e., the probability of falsely declaring a gene as DE, depending on the replicate number, with replicates of both conditions of the TOGE data (as for the power estimation) and with only biological replicates of a given condition (i.e., with, theoretically, no DE genes). All estimations were carried out with a threshold equal to 0.01 to control the FDR. For the former estimation, we pointed out the increase in the FPR with the replicate number, from about 1% with two replicates to 6% with seven replicates. To our knowledge, this drawback linked to the increase in the replicate number has not been underlined before in the literature. On the other hand, the results of the latter estimation show that the FPR is rather well controlled by the four studied methods (*DESeq, DESeq2*, and the two methods from *edgeR*). These results are in contradiction with those of Schurch et al. (2016), who found that *DESeq2* gave worse results than the other methods.

Another striking result that has not been shown yet in the literature, to our knowledge, is the impact of the threshold controlling the FDR on both the TPR and FPR. Indeed, by means of ROC curves depending on the threshold, we have shown that the optimal value for this threshold is almost equal to 2^−r^, where r is the replicate number. For instance, the optimal threshold is almost equal to 0.25 for two replicates, 0.12 for three replicates, 0.06 for four replicates, and so on. Obviously, as discussed before, this result has only been shown for our TOGE data, but the trend should still remain for other similar data sets. This result was shown for all four DE analysis methods studied. Moreover, we showed that for more than five replicates, the four methods give almost the same results, but, for fewer than five replicates, *DESeq* is slightly better than *DESeq2* and *edgeR* with the *exact test*, which are slightly better than *edgeR* with the *GLM* test.

We also performed a GO enrichment analysis depending on both the replicate number and library size. Such an analysis gives meaningful biological results in the sense that the measure is directly linked with the underlying biological processes. This analysis showed that the number of enriched categories (both true and false positive categories) increases significantly depending on the replicate number. On the contrary, the increase of depth does not significantly increase the number of enriched categories, but tends to decrease the rate of false positives. This new result is in adequacy with the trade-off between replicate number and library size discussed above for the number of DE genes and power.

As described above, a meaningful result of the present article comes from the meta-analysis that we made with all 17 projects on the tomato fruit extracted from the *TomExpress* platform and the TOGE data set. A descriptive analysis of the DE analyses performed within these projects clearly shows the impact of the replicate number and the library size, but also the distance between conditions and the variance of both conditions. Ching et al. (2014) underline the need for a high replicate number to accurately estimate the variance, and then obtain higher TPR and lower FPR. In the same way, Auer and Doerge (2010) also underline the need to properly estimate the variability. Nevertheless, our descriptive analysis shows that a huge variability still remains beyond the control of these known parameters. Moreover, we performed a more global analysis involving DE analyses between conditions of all 17 projects by simulating different samples with various library sizes (leading to 1,752,975 pairwise DE analyses). This meta-analysis showed that at least four replicates and 20 M reads are needed to be almost sure of obtaining about 1000 DE genes, no matter which biological conditions are studied. This meta-analysis also showed that, globally, a plateau is reached after about 10 replicates for all library sizes.

## Conclusion

As illustrated by the results above, we cannot *a priori* determine an optimal number of replicates for a given RNA-Seq experiment. Indeed, the statistical test used to perform a DE analysis, and then to declare a gene as significantly DE or not, depends not only on the replicate number and library size, but also on the distance between the biological conditions and on the variance of the given replicates. For example, it would not be surprising to find fewer DE genes between two close conditions of the tomato ripening process, such as Breaker+1 and Breaker+3 days, than between two distant conditions, such as Breaker+1 and Breaker+10 days. In a survey of best practices for RNA-Seq data analysis, Conesa et al. (2016) underline that, for a proper statistical power analysis, estimates of expression levels and dispersions of genes are required. That is why, in our opinion, the recommendations for RNA-Seq experimental designs should be moderated unless we take into account the percentage of wanted DE genes, the distance between conditions, the variance of these biological conditions, and so on.

Beyond the cost-effectiveness metric to guide the design of large scale RNA-Seq DE studies proposed by Liu et al. (2014), which also requires, *a priori*, all power values depending on replicate numbers and library sizes, we would advise RNA-Seq practitioners to use a pilot data set and dedicated tools to design their RNA-Seq experiments. If obtaining a pilot data set is not feasible, one can also use data sets that can be supposed to give almost similar parameters because, for instance, the studied biological conditions are similar. Nonetheless, some important further work would be the comparison of such existing tools. While we were writing our article, Poplawski and Binder (2017) proposed such a review of six tools for which they obtained widely different conclusions that seemed to be strongly affected by fold changes.

The results and discussion above will help RNA-Seq practitioners to better understand the impact of both replicate number and library size on a DE analysis, and also the impact of between-condition dispersion, which will help them to better design their experiments. For instance, we learned that choosing a threshold for FDR around 2^−r^ (with r the replicate number) should be optimal to enhance the sensitivity and specificity of the DE analysis. Moreover, for the RNA-Seq practitioners of the tomato community, the meta-analysis carried out in this study shows that at least four replicates and 20 M reads would be required to be almost sure of obtaining about 1000 DE genes, no matter which biological conditions they are interested in.

Ching et al. (2014) highlighted that no single software consistently showed the highest power across all the data sets they studied. We here recall that we have only performed our analyses with *DESeq, DESeq2*, and *edgeR*, which share common concepts, and that these R packages give, roughly speaking, similar results in the literature and in our study.

## Author contributions

EM and SL planed the research and performed statistical analyses; EM, MB, MZ, and PF designed the experiment of the TOGE project; DL and MZ performed the bioinformatics analyses of the TOGE project; ES performed statistical analyses; GH and PF performed the experiment of the TOGE project; VLB-A and MB advised on the analyses and interpretations of the results; and EM wrote the manuscript with contributions from all authors. All authors revised the work and the manuscript critically. All authors approved the final manuscript.

### Conflict of interest statement

The authors declare that the research was conducted in the absence of any commercial or financial relationships that could be construed as a potential conflict of interest.

## Footnotes

^1^https://www.bioinformatics.babraham.ac.uk/projects/fastqc

^2^https://github.com/happykhan/nfutil/blob/master/get_subset.py

^3^http://www.uniprot.org

^4^http://gbf.toulouse.inra.fr/tomexpress

## Supplementary material

Figure S1 Venn diagram of the number of genes declared as true DE with all four methods: *DESeq, DESeq2, edgeR GLM*, and *edgeR exact test*. The number of true DE genes that are common to all four methods is surrounded in blue. The number of true DE genes that are common to only *DESeq2* and both *edgeR* methods is surrounded in red.

Figure S2 First species risk curves depending on the depth **(A)** and on the number of replicates **(B)**. The DE analysis method used here is *edgeR exact test* as for both the number of DE genes and power analyses (see Material and methods).

Figure S3 Estimations of FPR for the four studied DE analysis methods. A zoomed version of Figure 3B.

Figure S4 ROC curves for the *DESeq* method.

Figure S5 ROC curves for the *edgeR GLM* method.

Figure S6 ROC curves for the *edgeR exact test* method.

Figure S7 Optimal values of the FDR parameter for the four studied methods, *DESeq, DESeq2, edgeR GLM*, and *edgeR exact test*, for each replicate number r from 2 to 7, are represented by black crosses. These values are extracted from ROC curves of Figure 4 and Figures S4–S6. Red dots and line represent the fitted curve f depending on the number of replicates r. The curve f has been fitted by a linear regression of the log_2_ optimal values (dependent variable) depending on the number of replicates (explanatory variable).
