# Supplementary figures and images for: Optimization of an RNA-Seq Differential Gene Expression Analysis Depending on Biological Replicate Number and Library Size
Source: Front Plant Sci. 2018 Feb 14;9:108. doi: 10.3389/fpls.2018.00108 (PMC5817962; doi:10.3389/fpls.2018.00108)

## Slide 1
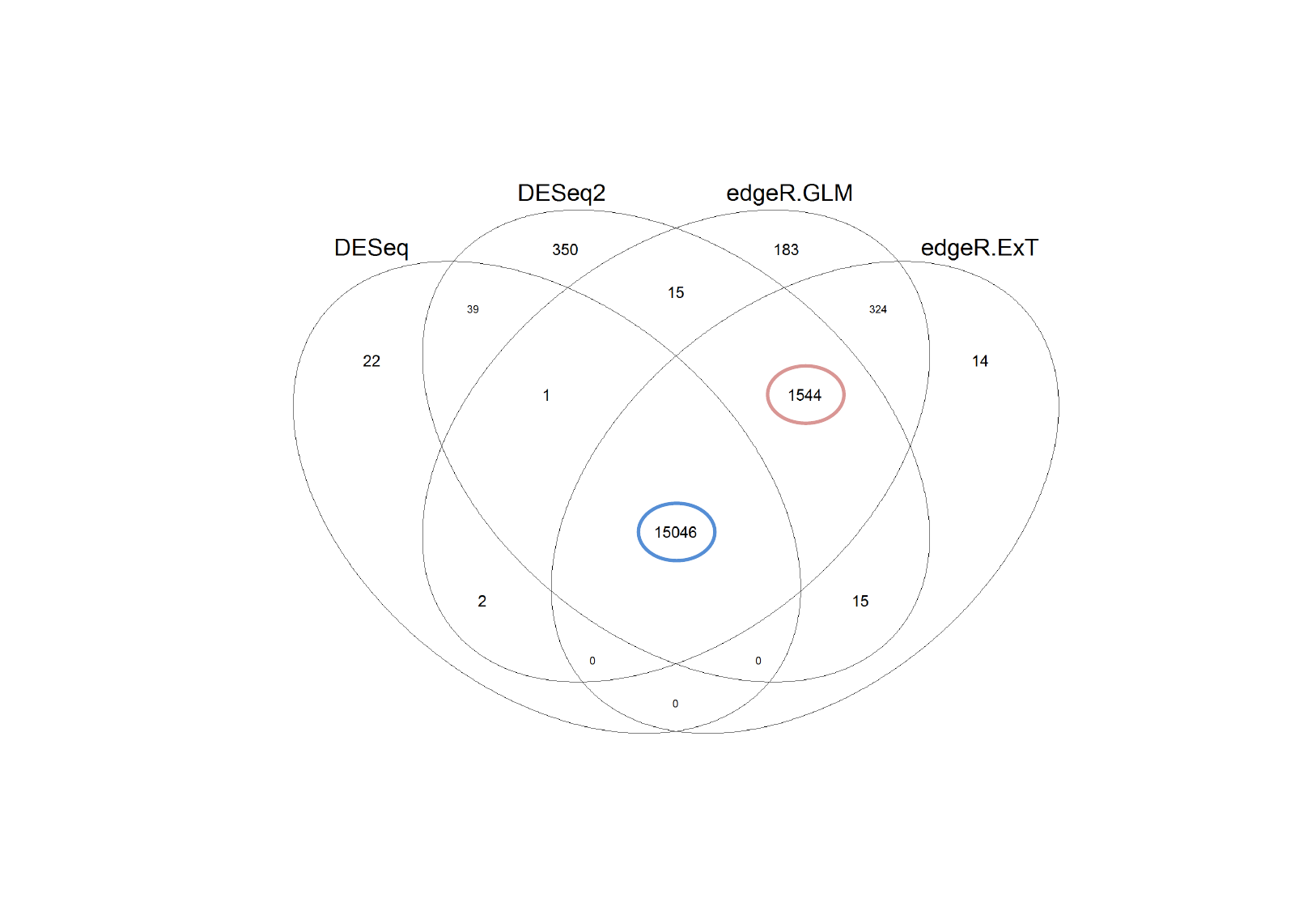

## Slide 2
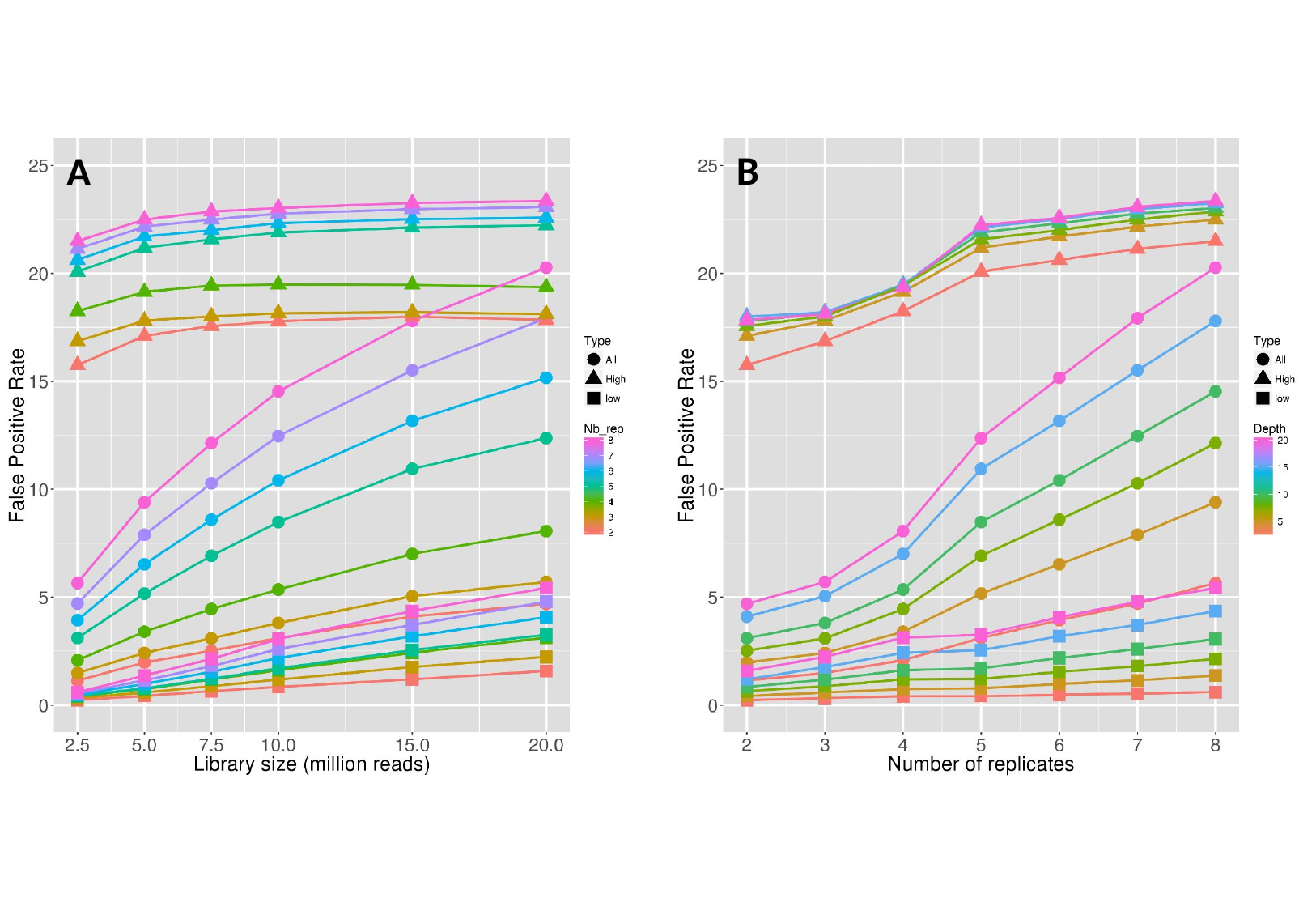

B
A

## Slide 3
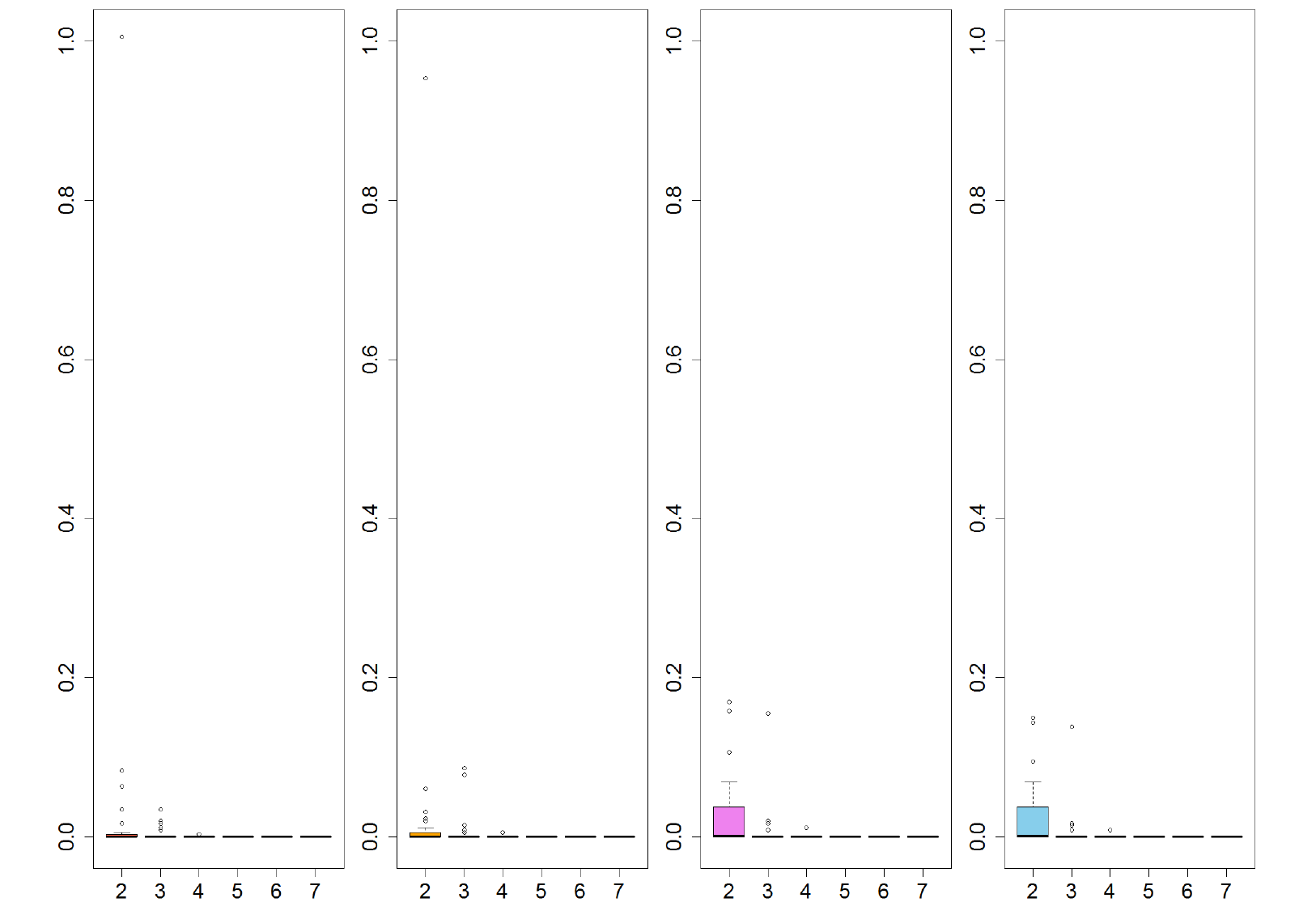

## Slide 4
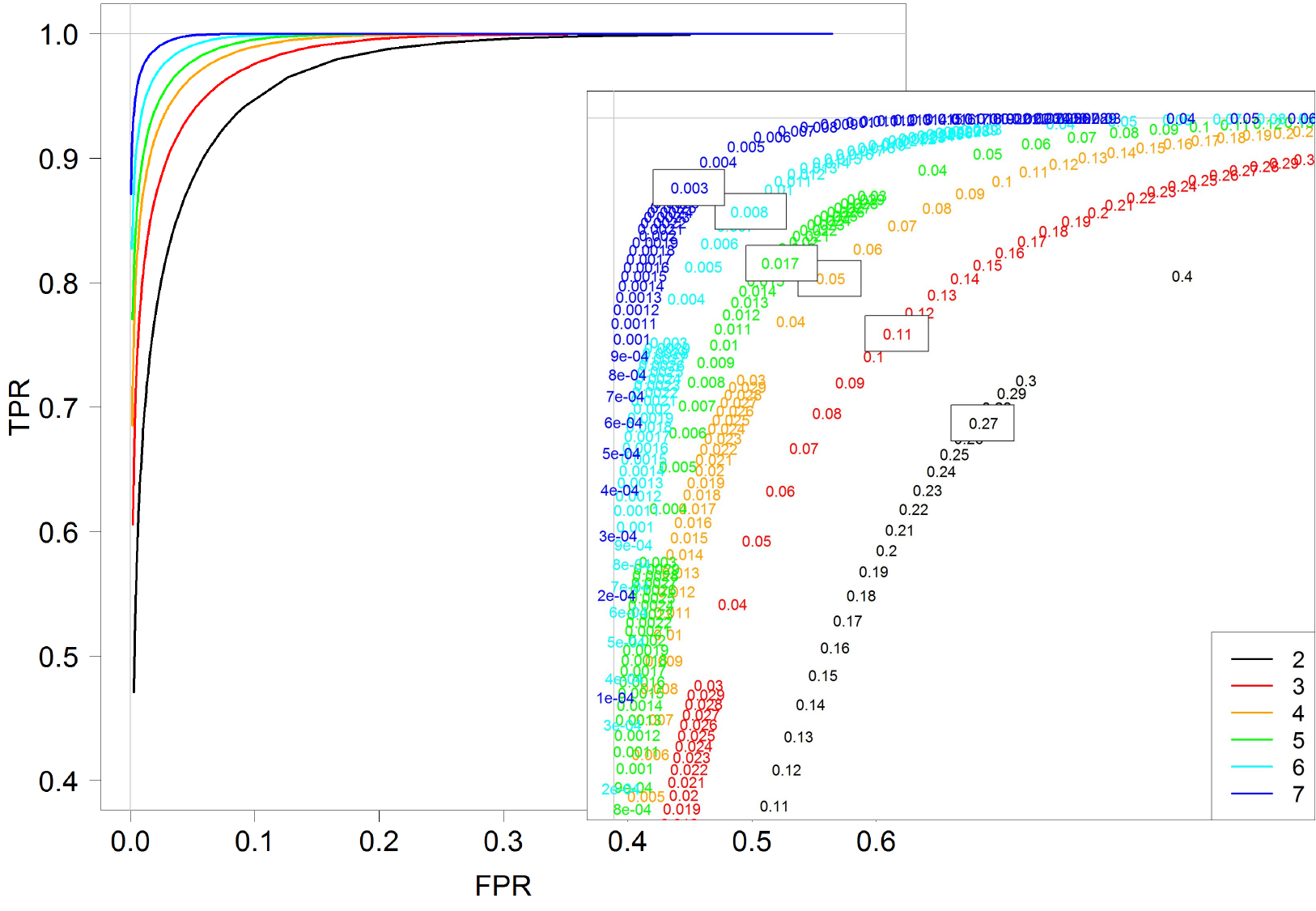

## Slide 5
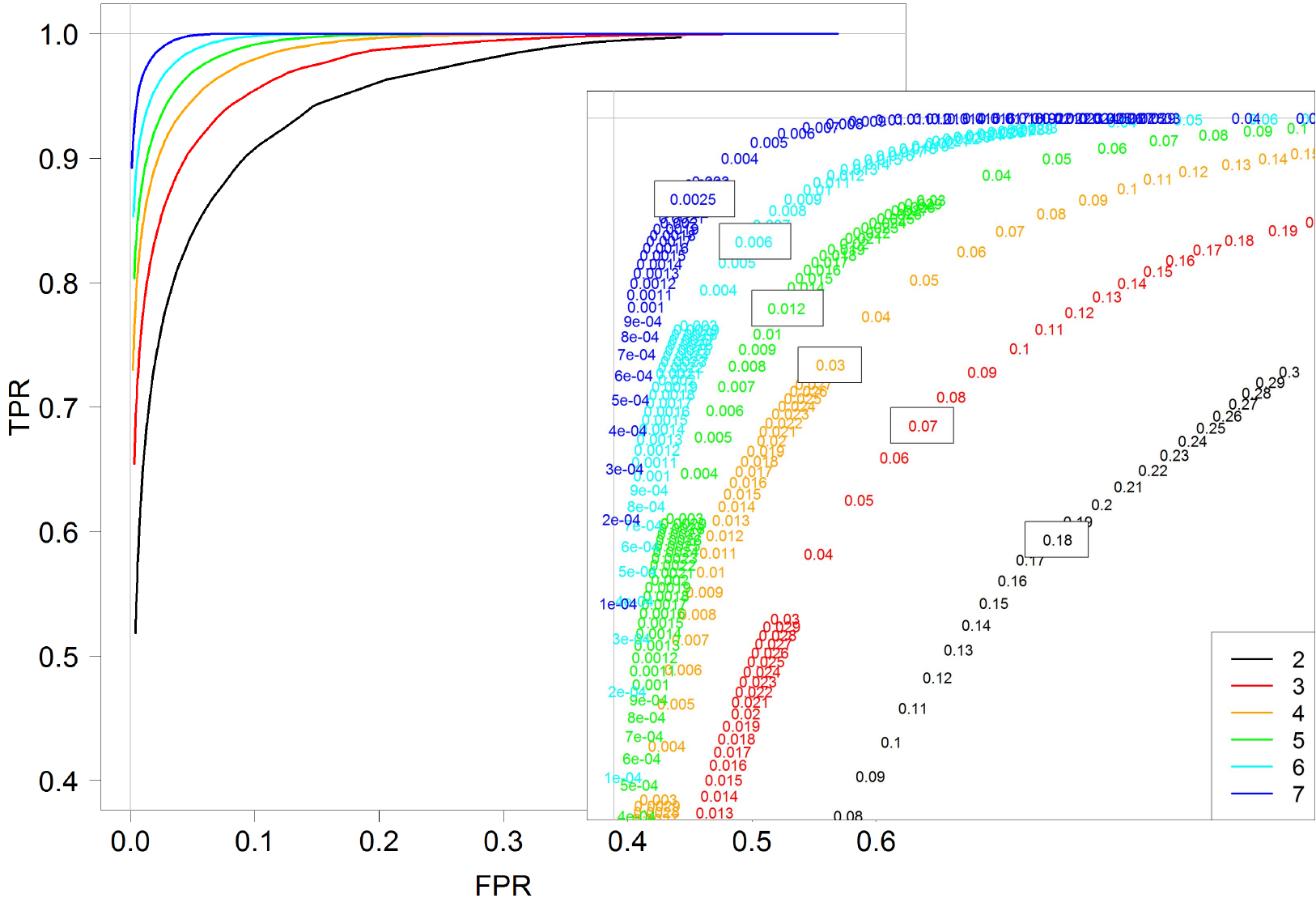

## Slide 6
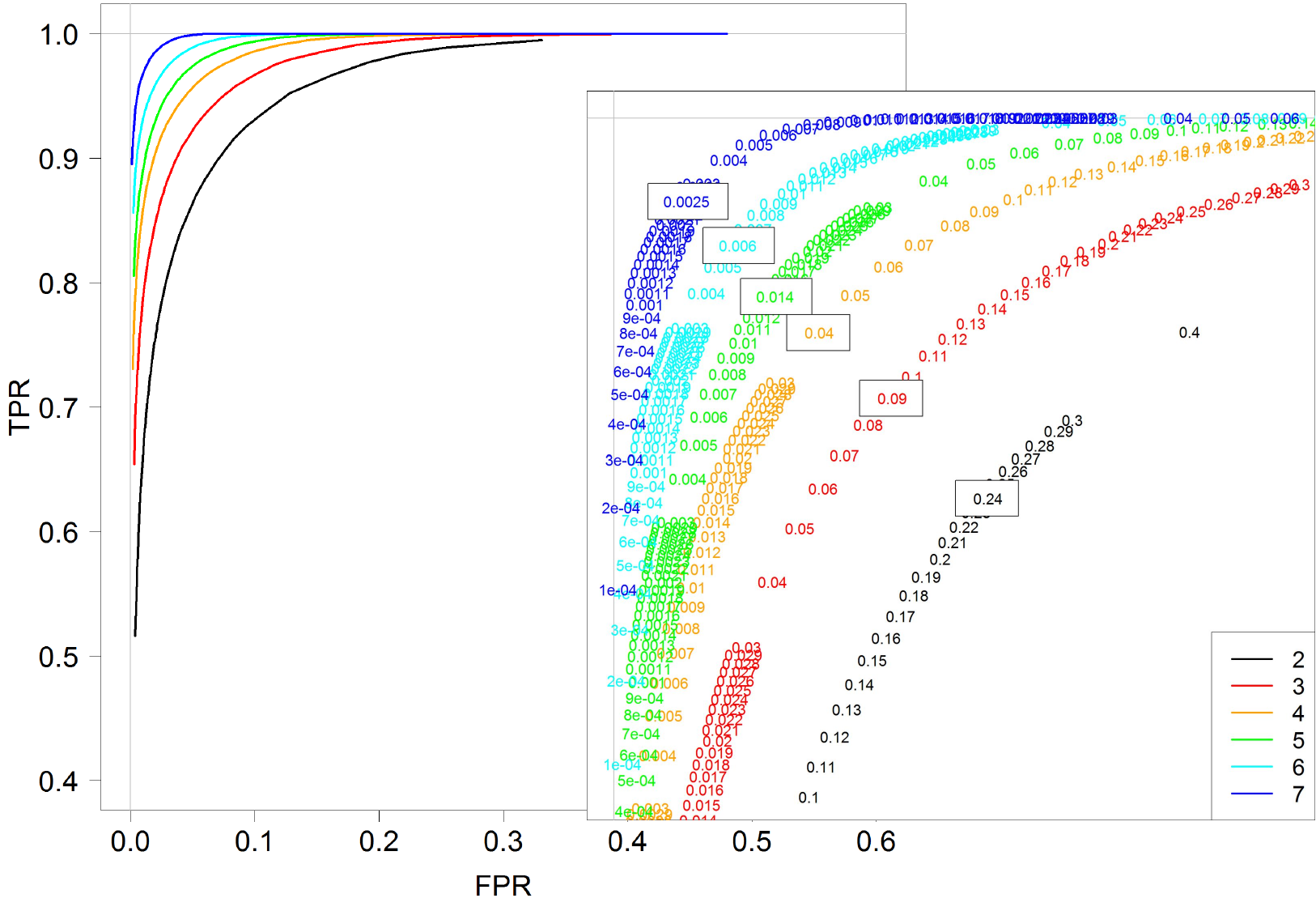

## Slide 7
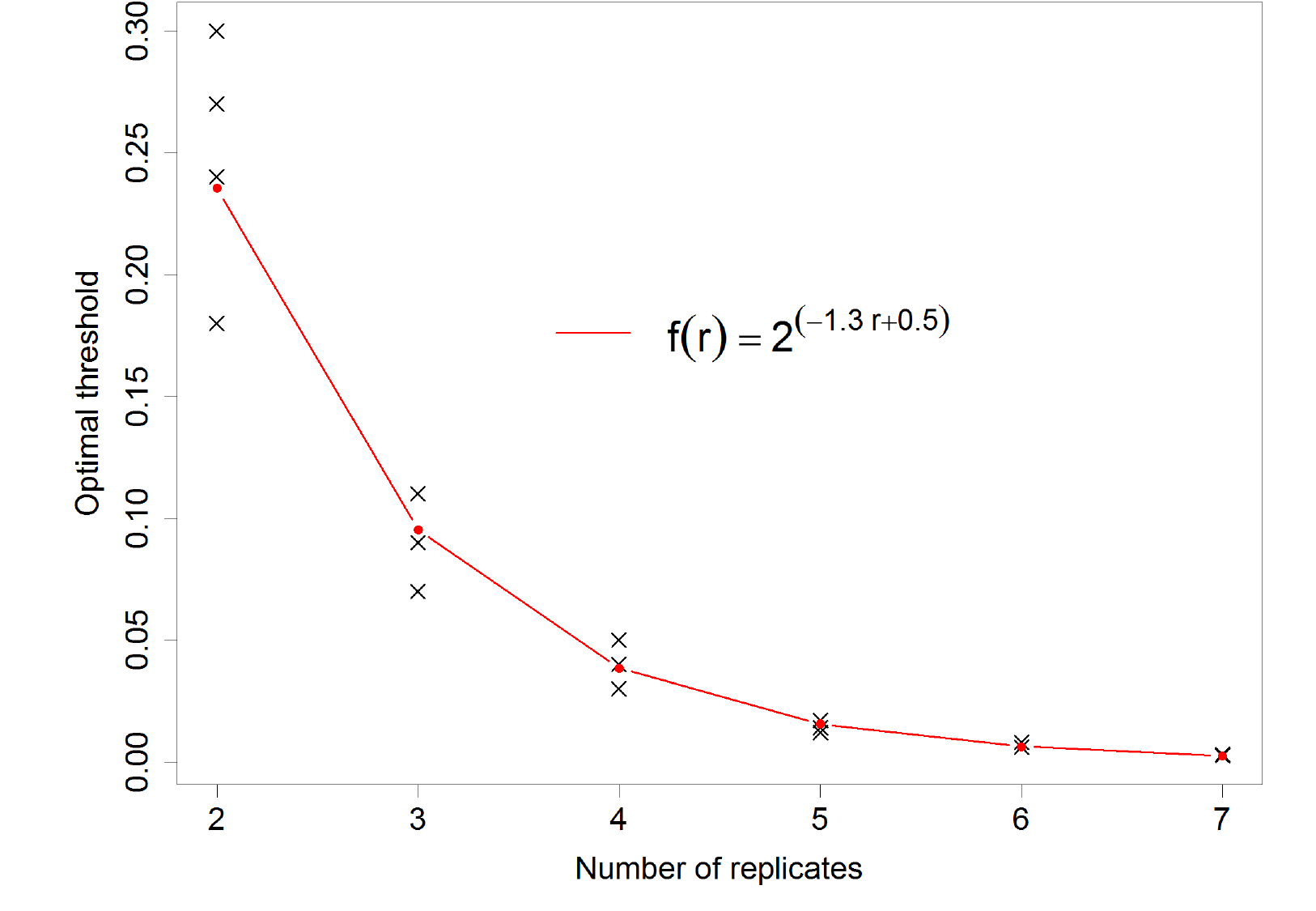

Supplement: Figure S1 — Venn diagram of the number of genes declared as true DE with all four methods: DESeq, DESeq2, edgeR GLM, and edgeR exact test. The number of true DE genes that are common to all four methods is surrounded in blue. The number of true DE genes that are common to only DESeq2 and both edgeR methods is surrounded in red. [file Presentation1.PPTX]
